# Tackling abiotic stress in plants: recent insights and trends

**DOI:** 10.1007/s44154-025-00216-x

**Published:** 2025-01-26

**Authors:** Heng Zhang, Zhaobo Lang, Jian-Kang Zhu, Pengcheng Wang

**Affiliations:** 1 Department of Genetics and Developmental Science, School of Life Sciences and Biotechnology, Shanghai Jiao Tong University, Shanghai, China; 2 Institute of Advanced Biotechnology and School of Medicine, Southern University of Science and Technology, Shenzhen, 518055 China; 3 Institute of Advanced Biotechnology, Institute of Homeostatic Medicine, and School of Medicine, Southern University of Science and Technology, Shenzhen, 518055 China

**Keywords:** Abiotic stress, Calcium signaling, Liquid–liquid phase separation, Protein modification, Crop resilience, Sustainable agriculture, Stress adaptation

## Abstract

Plants, as sessile organisms, must adapt to a range of abiotic stresses, including drought, salinity, heat, and cold, which are increasingly exacerbated by climate change. These stresses significantly impact crop productivity, posing challenges for sustainable agriculture and food security. Recent advances in omics studies and genetics have shed light on molecular mechanisms underlying plant stress responses, including the role of calcium (Ca^2^⁺) signaling, liquid–liquid phase separation (LLPS), and cell wall-associated sensors in detecting and responding to environmental changes. However, gaps remain in understanding how rapid stress signaling is integrated with slower, adaptive processes. Emerging evidence also highlights crosstalk between abiotic stress responses, plant immunity, and growth regulation, mediated by key components such as RAF-SnRK2 kinase cascades, DELLA proteins, etc. Strategies to enhance crop stress resistance without compromising yield include introducing beneficial alleles, spatiotemporal optimization of stress responses, and decoupling stress signaling from growth inhibition. This review emphasizes the importance of interdisciplinary approaches and innovative technologies to bridge fundamental research and practical agricultural applications, aiming to develop resilient crops for sustainable food production in an era of escalating environmental challenges.

## Introduction

As sessile organisms, plants must continually adapt to ever-changing environments. A significant portion of the Earth's arable land faces challenging environmental stress conditions like droughts, heat waves, and flooding for plant growth. Climate change exacerbates these stresses, with significant consequences on plant growth, development and productivity (Terán et al. 2024; Ahuja et al. 2010; Benitez-Alfonso et al. 2023). Abiotic stresses can reduce crop yields by over 50% on average compared to optimal conditions (Kopecká et al. 2023; Calanca 2017). Understanding how plants sense and respond to stress conditions, pinpointing key genes essential for stress adaptation, and cultivating stress-resistant crops have become imperative endeavors for sustainable agriculture and ensuring global food security. Recent advances in omics studies and genetics have revealed numerous genes important for abiotic stress responses, deepening our understanding of plant adaptation mechanisms. However, identifying sensors for physical stressors remains a complex challenge compared to discerning chemical ligand receptors. Moreover, enhancing stress resilience diverts cellular resources and often leads to growth inhibition, making it very difficult to boost crop yields under stress conditions. In this concise review, we aim to highlight recent advances, recognize current trends, and provide some insights into future directions in the field of plant abiotic stress biology.

**Fig. 1:**
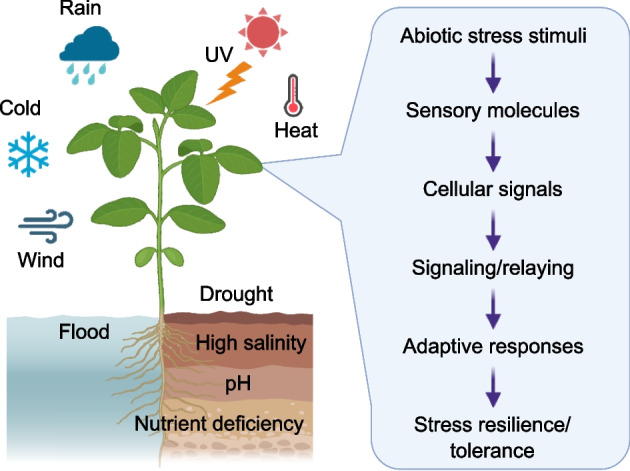
A conceptual model for plant sensing and adaptation to environmental changes. Specialized sensory molecules located in various cellular compartments—such as the cell wall, cytosol, and organelles—detect environmental changes and translate these stimuli into specific cellular signals. These secondary signals coordinate diverse cellular processes, including changes in gene expression, vesicle trafficking, metabolism, stomatal dynamics, and growth, enabling plants to adapt to fluctuating environmental conditions

Typically, specialized sensory molecules detect environmental changes in various cellular compartments such as the cell wall, cytosol, and organelles. Upon detection, these stress sensors undergo physical changes, which affects their enzyme activities or interactions with other cellular components, thereby translating physical stressors into specific cellular chemical signals. These signals often manifest as second messengers or post-translational modifications (PTMs) of proteins. These secondary signals orchestrate a series of cellular processes—including changes in gene expression, vesicle trafficking, metabolism, stomatal opening, and growth and development, enabling plants to adapt to diverse environmental fluctuations (Fig. 1).

## Sensing mechanism for environmental stimuli

**Fig. 2:**
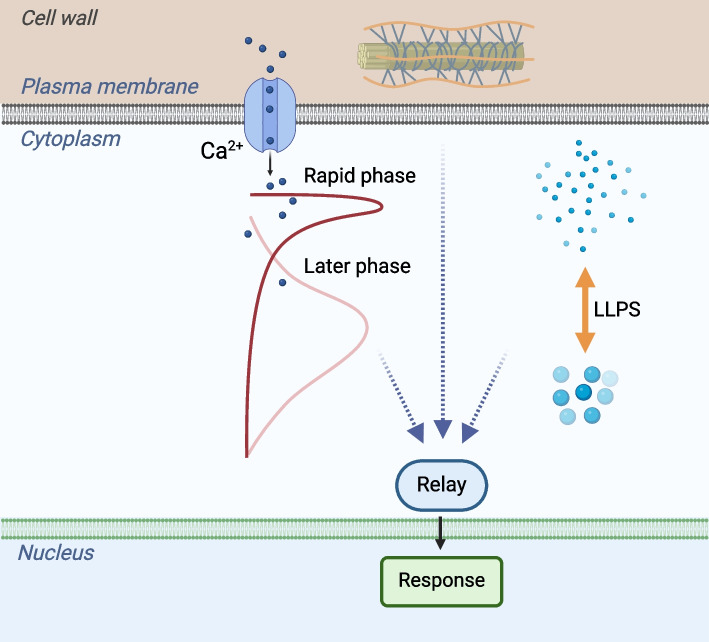
Central roles of calcium signaling, LLPS, and the cell wall components in sensing and relaying abiotic stresses. Emerging evidence highlights the significance of Ca^2^⁺ signaling, liquid–liquid phase separation (LLPS), and cell wall components in plant responses to diverse environmental changes. While the integration of signals from these sensory mechanisms remains unclear, it is an important area for future exploration. Stress responses occur in various cellular regions; this figure focuses on the nucleus as a representative example

Calcium (Ca^2+^) is a second messenger in plants, playing important roles in the signaling of various stresses (Gorgues et al. 2022) (Fig. 2). Several proteins have been reported to be involved in the sensing of abiotic stresses such as hyperosmotic stress, Na^+^, and chilling stress, because mutations in them affect cytosolic Ca^2+^ levels under stress. The Arabidopsis *osca1* (hyperosmolality-gated calcium-permeable channel) mutant, identified through an aequorin-based Ca^2+^ measurement system, exhibited impaired cytosolic Ca^2+^ increases in response to sorbitol treatment (Yuan et al. 2014). The plant OSCA proteins share structural similarities with animal TMEM63 proteins, recently recognized as inherently mechanosensitive channels involved in various functions such as food texture sensing in insects, and hearing and brain functions in mice (Murthy et al. 2018; Jojoa-Cruz et al. 2018). The same mutant screen identified the monocation-induced [Ca^2+^]_i_ increases 1 (MOCA1), a glucuronosyltransferase for glycosyl inositol phosphorylceramide (GIPC) synthesis, and a potential sensory mechanism for sodium chloride (Jiang et al. 2019), and HPCA1, a plasma membrane-localized protein kinase as a candidate sensor for apoplastic hydrogen peroxide (Wu et al. 2020). HPCA1 is also a potential receptor for the host-derived quinone compound 2,6-dimethoxy-1,4-benzoquinone (DMBQ) (Laohavisit et al. 2020). Recently, the homologues of OSCA1, OSCA2.1 and OSCA2.2, were reported to be potential sensors of hypoosmolarity, since the Arabidopsis double mutant *osca2.1/2.2* was impaired in pollen germination (Pei et al. 2024). In rice, COLD1, a regulator of G-protein signaling, was reported as a cold stress sensor that regulates Ca^2+^ influx under low temperature conditions (Ma et al. 2015). Despite the critical roles of these proteins in Ca^2+^ signaling in response to various abiotic stresses, their downstream effectors and physiological functions in abiotic stress adaptation remain largely unclear. For instance, while the RAF-SnRK2 protein kinase cascade is known to be rapidly activated by hyperosmolarity and serves as central regulatory module in plant osmotic stress responses, its activation appears unaffected in high-order mutants of *OSCAs* (Lin et al. 2020), even though it is activated by the calcium chelator EGTA (Sang et al. 2024). SnRK2s are known to phosphorylate cyclic nucleotide-gated channel 5 (CNGC5) as well as CNGC6, CNGC9 and CNGC12 at a conserved serine residue to orchestrate ABA-activated cytosolic Ca^2+^ oscillations and Ca^2+^ currents in guard cells (Yang et al. 2024). In addition, visualizing ABA-triggered Ca^2+^ dynamics in the root cap revealed that ABA-triggered Ca^2+^ increase is largely dependent on ABA receptors and SnRK2.2/3/6 (Lin et al. 2024). Similarly, the cold-activated receptor-like protein kinase PLANT PEPTIDE CONTAINING SULFATED TYROSINE1 RECEPTOR (PSY1R) phosphorylates CNGC20 to mediate cold evoked Ca^2+^ influx and freezing tolerance (Peng et al. 2024). Thus, these environmental stimuli likely evoke at least two phases of cytosolic Ca^2+^ increases: a rapid early phase and a later phase. The rapid phase of Ca^2+^ increase, which is evoked in seconds, is largely dependent on the sensory channels (e.g., OSCA1, OSCA2 and MSLs) or regulators of channels (e.g., COLD1, BON1, MOCA1, and HPCA1/CARD1). However, the calcium channels regulated by COLD1, MOCA1, and HPCA1/CARD1 to control Ca^2+^ influx remain elusive. The downstream effectors that decode these rapid Ca^2+^ increases to regulate adaptive processes need to be identified. Environmental stimuli also evoke cytosolic Ca^2+^ increases at a later phase, which occurs in minutes or oscillates in hours after stimulation. This later phase of Ca^2+^ increases likely depends on some well-characterized signaling components, like SnRK2s in ABA and osmotic stress response pathways, to phosphorylate and activate CNGCs or other Ca^2+^ channels (Yang et al. 2024). Besides phosphorylation, other PTMs, interactions with other proteins (e.g., Calmodulin), and small molecules (e.g., cGMP) may also participate in the activation of Ca^2+^ channels in the later phase, which is worthy of study in the future. The signaling components bridging the rapid and later phases of cytosolic Ca^2+^ increases are the major gaps to be addressed in calcium signaling in abiotic stress responses.

In recent years, liquid–liquid phase separation (LLPS) has emerged as a critical mechanism for plants to sense and respond to environmental changes (Fig. 2) (Wang and Zhu 2025; Liu et al. 2023). For instance, in Arabidopsis seeds, the FLOE1 protein acts as a potential water sensor, inhibiting germination under unfavorable conditions (Dorone et al. 2021). Upon hydration, these FLOE1 molecules form condensates, facilitating embryo release and germination. Similarly, the transcriptional regulator SEUSS (SEU) undergoes nuclear condensation in response to hyperosmotic stress, thereby modulating osmotic stress-responsive gene expression. Recently, DCP5 was reported to sense molecular crowding caused by hyperosmolarity (Wang et al. 2024c, 2024b). FREE1, a well-characterized substrate of SnRK2s, condenses upon multiple forms of osmotic stress and mediates the bending and scission of endosome membranes (Wang et al. 2024d). Additionally, LLPS-driven biomolecular condensates are also induced by ambient temperatures. Proteins like phytochrome B (phyB), ELF3, and RNA-binding glycine-rich proteins D2 and D4 (RBGD2/RBGD4) undergo LLPS, enabling plants to sense variations in ambient temperature or combinations of temperature and light (Jung et al. 2016). TWA1, a transcriptional co-regulator, undergoes LLPS upon exposure to elevated temperatures (Bohn et al. 2024). At elevated temperatures, TWA1 accumulates in nuclear subdomains, and physically interacts with JASMONATE-ASSOCIATED MYC-LIKE (JAM) transcription factors and with TOPLESS (TPL) and TOPLESS-RELATED (TPR) for repressor complex assembly (Bohn et al. 2024). These LLPS-driven biomolecular condensates could be composed of different types of macromolecules—including proteins, nucleic acids, and lipids—enabling nuanced regulation of cellular processes like signal transduction, cargo sorting, and gene transcription. Notably, LLPS is not limited to intracellular environments; recent research has highlighted LLPS of extracellular components like pectin and RALF peptides (Liu et al. 2024). Thus, LLPS may represent a universal and reversible sensory mechanism to detect changes in cellular osmolarity (e.g., salinity or dehydration) and temperature, enabling plants to perceive environmental cues and coordinate a wide array of biological responses (Liu et al. 2023).

While the pivotal roles of Ca^2+^ signaling and biomolecular condensation in abiotic stress sensing and signaling are well appreciated, the connection between them remains largely uncharted. Increased cytosolic [Ca^2+^] may activate protein kinases and other enzymes mediating PTMs. PTMs, including methylation, ubiquitylation, SUMOylation, and phosphorylation, have been shown to regulate biomolecular phase separation. Conversely, phase separation can modulate the activity of PTM enzymes and, consequently, the PTM of target proteins. Plant cells may employ similar molecular mechanisms to synchronize membrane-associated signaling with liquid–liquid phase separation (LLPS) in abiotic stress responses. Plant cells are enveloped by cell walls, which play crucial roles in sensing environmental cues and transmitting stress signals to the cell membrane and interior (see (Colin et al. 2023) for a review). Notably, FERONIA (FER), a cell wall-associated and plasma membrane-localized Catharanthus roseus receptor-like kinase 1-like (CrRLK1L) protein, interacts with its ligands—RALF peptides and cell wall-localized LRR extensin (LRX) proteins—to monitor cell wall perturbations. Under stress conditions, cell surface pectin-RALF1 phase separation recruits FERONIA-LLG1 into condensates (Liu et al. 2024). FER likely promotes Ca^2+^ influx, thereby triggering various cellular responses (Colin et al. 2023). Other components of the cell wall, such as the cell wall integrity (CWI) surveillance system and cellulose synthase (CESA) complexes (CSCs), also play important roles in sensing and responding to abiotic stresses (Colin et al. 2023). Investigating how signals from the cell wall, the plasma membrane, and the interior are integrated to coordinate stress responses is a topic deserving further study in the future.

## Cross-talk between abiotic, biotic stress responses and growth/development

Emerging evidence highlights a close relationship between abiotic and biotic stress responses in plants. For instance, key regulators in plant immunity, Ca^2+^-responsive phospholipid-binding BONZAI (BON) proteins, were found to play a crucial role in global osmotic stress responses (Chen et al. 2020). A host and microbe-derived small peptide PEP1, along with its kinase receptor PEPR1, function as sensors for apoplastic pH (Liu et al. 2022a). The RALF-FER module serves as a central orchestrator of both immunity and abiotic stress signaling. The secreted peptides SMALL PHYTOCYTOKINES REGULATING DEFENSE AND WATER LOSS (SCREWs) and their cognate receptor kinase, PLANT SCREW UNRESPONSIVE RECEPTOR (NUT), counterbalance ABA- and microbe-associated molecular pattern (MAMP)-induced stomatal closure (Liu et al. 2022b). Furthermore, the Arabidopsis LRR-RLK BRI-ASSOCIATED RECEPTOR KINASE 1 (BAK1, also referred to as SOMATIC EMBRYOGENESIS RECEPTOR-LIKE KINASE 3, SERK3) not only functions as a co-receptor in responses to pathogenic bacterial attacks but also plays a role in drought stress response and ABA signaling. Botrytis-induced kinase 1 (BIK1), a receptor-like cytoplasmic kinase that participates in the perception and signaling of multiple pathogen effectors, is reported to phosphorylate SnRK2s upon osmotic stresses (Li et al. 2024). Phosphorylation of BIK1 at two conserved tyrosine residues in the activation loop of SnRK2s prevents the binding and dephosphorylation activity of PP2Cs. Interestingly, bacteria utilize the osmotic stress and abscisic acid signaling pathways to induce stomatal closure, maintaining the necessary apoplast humidity for bacterial growth. These studies suggest a universal interplay between abiotic stress signaling and immunity. One plausible explanation for this interplay is that during evolution—especially as ancient plants transitioned from water to land—they likely repurposed the already well-established immunity signaling components to respond to increasingly severe and rapidly fluctuating environmental challenges.

The intricate relationship between abiotic stress signaling and growth and development pathways is noteworthy. The DELLAs are plant-specific proteins that negatively regulate gibberellin (GA) signaling immediately downstream of the GA receptor (Achard et al. 2006). The DELLA proteins also participate in salt stress signaling (Achard et al. 2006). Under normal conditions, the Target of Rapamycin (TOR) phosphorylates ABA receptors, suppressing stress signaling. Environmental stresses like drought rapidly activate SnRK2s, which phosphorylate RAPTOR1 to inhibit TOR signaling, subsequently impacting plant growth and development (Wang et al. 2018). Furthermore, SnRK1s, pivotal regulators in sugar and nutrient signaling, may also play a role in suppressing TOR signaling under stress conditions (Belda-Palazon et al. 2020). A recent study highlighted that RAF kinases, upstream activators of SnRK2s, undergo hyperphosphorylation within seconds of auxin application and mediate rapid auxin responses (Kuhn et al. 2023). The RAF kinases likely serve as evolutionarily conserved regulators that balance stress signaling with growth. They are involved in a myriad of signaling pathways, including auxin, ABA, ethylene, drought, hyperosmolarity, flooding, potassium, and immunity signaling, from moss (*Physcomitrella patens*) to land plants (Arabidopsis) (Kuhn et al. 2023; Wang 2024b, a). These and other as yet to be discovered mechanisms coordinate plant growth and stress adaptation signaling, enabling plants to efficiently respond to fluctuating environmental conditions.

## Genetic strategies for stress resilience plants

Stress response and acclimation are evolutionarily coupled with growth inhibition. Therefore, enhancing crop stress resistance without compromising growth and yield is a formidable challenge. Nevertheless, several studies reported potentially successful stories, pointing toward strategies to address this challenge. Firstly, introducing superior alleles of specific genes related to abiotic stress tolerance was shown to enhance stress resistance in crops. For instance, a single SNP in COLD1 increases cold tolerance in rice (Ma et al. 2015). Knockout mutants of AT1/GS3, a Gγ subunit, exhibit improved alkaline tolerance and increased yield in monocotyledonous crops like rice, sorghum, millet, and maize (Zhang et al. 2023). Interestingly, a single nucleotide mutation in the same gene (referred to as *THERMOTOLERANCE 2*, *TT2*), which leads to premature translation, also enhances thermotolerance in rice by stabilizing wax biosynthesis during heat stress (Kan et al. 2022). Modulating the expression of *THERMOTOLERANCE 3.1* (*TT3.1*) or knocking out *TT3.2* can enhance rice heat tolerance without affecting yields in the field (Zhang et al. 2022). Secondly, enhancing stress response with spatiotemporal specificity might increase crop stress tolerance without significant yield penalty. In recent years, certain ABA-mimicking chemicals, such as opabactin (OP) (Vaidya et al. 2019) and AMFs (Cao et al. 2017), have shown promise in enhancing crop stress tolerance. Introducing the bacterial light-gated K^+^ channel BLINK1 into Arabidopsis guard cells accelerates stomatal responses to light, improving water use efficiency without compromising carbon fixation, thus increasing biomass in transgenic plants (Papanatsiou et al. 2019). Thirdly, decoupling stress signaling from plant growth could boost yields under stress conditions. For instance, mutating three rice ABA receptors —PYL1, PYL4, and PYL6— led to yield increases of up to 25% without affecting field performance in paddy rice (Miao et al. 2018). A recent study showed that engineering source–sink relations by prime editing confers resilience to heat stress in both tomato and rice (Lou et al. 2024). Harnessing root-associated microbes offers another avenue to promote crop resistance and potentially increase yields under stress (de Vries et al. 2020; Allsup et al. 2023). However, the application of these genetic strategies in agricultural settings necessitates further rigorous validation and integration with holistic farming approaches. As commented in a recent article (Khaipho-Burch et al. 2023), although crop yield can be affected by some single genes, these genes must function with many other genes involved in domestication and adaptation, and in the context of appropriate soil and fertilizer management regimes.

## Conclusion and perspectives

In conclusion, the intricate landscape of plant responses to abiotic stresses reveals a sophisticated interplay of molecular mechanisms, genetic pathways, and environmental cues. While significant progress has been made in understanding these complexities, much remains to be elucidated, particularly regarding the integration of sensing mechanisms and various signaling networks and the strategies to improve stress resistance without affecting plant productivity. Research findings in the lab also require significant effort to be translated into practical agricultural applications. The multifaceted nature of stress resistance—spanning cellular responses and ecosystem interactions—underscores the need for a holistic approach that encompasses genetics, physiology, agronomy, chemical and microbial interventions, and environmental science. As we advance into an era marked by escalating environmental challenges and growing food demand, leveraging interdisciplinary collaborations, innovative technologies such as artificial interlligence, and sustainable practices will be essential. Ultimately, enhancing crop stress resistance without compromising yield represents not only a scientific endeavor but also a critical pathway toward ensuring global food security, ecological sustainability, and resilience in the face of an uncertain future.

## Data Availability

Not applicable.

## Abbreviations

ABA: Abscisic acid
Ca^2+^: Calcium
GA: Gibberellin
LLPS: Liquid-liquid phase separation
